# Association between dietary patterns and infant birth weight in brazilian pregnancy women with gestational diabetes: a cross-sectional study

**DOI:** 10.61622/rbgo/2024rbgo68

**Published:** 2024-09-06

**Authors:** Luciana da Cunha Bernardes Argenta, Nadya Helena Alves dos Santos, Cláudia Saunders, Joana Dias da Costa, Letícia Victoria Souza da Cunha, Pamela Melo Krok Fedeszen, Patricia de Carvalho Padilha

**Affiliations:** 1 Universidade Federal do Rio de Janeiro Rio de Janeiro RJ Brazil Universidade Federal do Rio de Janeiro, Rio de Janeiro, RJ, Brazil.

**Keywords:** Dietary patterns, Diabetes, gestational, Birth weight, Infant, newborn, Pregnant women

## Abstract

**Objective:**

To evaluate the association between the dietary patterns (DPs) of pregnant women with GDM (gestational diabetes *mellitus*) and the birth weight (BW) of the infants.

**Methods:**

Cross-sectional study with 187 adult pregnant women with GDM attended at a maternity in Rio de Janeiro from 2011 to 2014. Dietary intake was assessed in the third trimester using a semiquantitative food frequency questionnaire (FFQ). The outcomes were BW and weight adequacy for gestational age (GA). Reduced Rank Regression (RRR) was used to explain the following response variables: density of carbohydrates, fibres, and saturated fatty acids. Statistical analyzes included multinomial logistic regression models.

**Results:**

The mean BW was 3261.9 (± 424.5) g. Three DPs were identified, with DP 3 (high consumption of refined carbohydrates, fast foods/snacks, whole milk, sugars/sweets, and soft drinks and low consumption of beans, vegetables, and low-fat milk and derivatives) being the main pattern, explaining 48.37% of the response variables. In the multinomial logistic regression analysis no statistically significant association was found between the tertiles of DPs and BW or the adequacy of weight for GA, even after adjustments of confounding covariates.

**Conclusion:**

No significant associations were found between maternal DPs in the third trimester of pregnancy and infant BW or adequacy of weight for GA.

## Introduction

Gestational diabetes mellitus (GDM) is defined by the World Health Organization as a carbohydrate intolerance of varying severity that begins during a current pregnancy and does not meet the diagnostic criteria of frank diabetes mellitus (DM). It is the most common metabolic problem in pregnant women and has a prevalence of 3–25% of all pregnancies, depending on the ethnic group, population, and diagnostic criterion used.^1^

This pathology is usually diagnosed in the second or third trimester of pregnancy and can have negative consequences for both the mother and fetus or neonate.^1^ The risk of GDM increases with the progression of hyperglycaemia.^2^

Inadequate fetal development is an important consequence of GDM. Birth weight (BW) is a parameter used to assess the health conditions of newborns. It is an indicator of the initial development of life and is associated with a series of immediate, short-term, and long-term outcomes.^3^

Low BW (< 2.5 kg) is a determining factor of fetal and neonatal mortality and morbidity and cognitive developmental deficits.^4^ In the other extreme, fetal macrosomia (BW ≥ 4 kg), an important perinatal concern because it occurs in 15–45% of babies born to mothers with DM,^5^ is related to adverse outcomes such as neonatal asphyxia, increased risk of fetal hypoglycaemia, premature labour, fetal–pelvic disproportion, skeletal trauma, and hydro-electrolytic disorders.^6,7^ Further, in the long term, inadequate fetal growth favours the development of chronic non-communicable diseases in adulthood.^7^

Lifestyle factors before and during pregnancy, including nutritional factors, are important in the pathogenesis of GDM or in the course of the disease and can have a great impact on maternal and perinatal outcomes.^8^ In most cases, nutritional therapy, physical activity, and weight management can sufficiently control GDM.^2^

Maternal nutritional status, along with placental function and genetic potential, is the main contributor to infant BW.^9^ The overall quality of maternal diet including the intake of essential nutrients is closely linked to pregnancy outcomes such as BW and its relationship to gestational age (GA); however, these associations are still unclear.^10^

Although several studies have focused on the investigation of nutrients or isolated foods, these are consumed in combination with other dietary components and the individual effect is difficult to determine. In this sense, analysis of the dietary patterns (DPs), which represents a set of inter-related dietary factors, is an important approach to evaluate both the diet–GDM^8^ and maternal diet–perinatal outcome^11^ relationships; however, only a few studies have been conducted, with conflicting results.

Therefore, the DPs are related to the development and control of GDM, and may also reflect on BW, which can influence health throughout life. Taking into consideration this fact, the aim of this study was to evaluate the association between the DPs of pregnant women with GDM and the BW of their infants.

## Methods

This cross-sectional study nested to a longitudinal study used information from a database that is part of a project titled ‘Theoretical–Practical Contributions to Prenatal Care of Diabetic Pregnant Women’. The study population comprised pregnant women with GDM and their newborns who attended the prenatal and nutrition service of a public maternity hospital in the city of Rio de Janeiro, during pregnancy, delivery, and the immediate postpartum period, between 2011 and 2014.

For the construction of the sample from the original database, the following inclusion criteria were considered: (i) diagnosis of GDM made or confirmed in the unit,^12^ (ii) GA up to 28 weeks according to the date of last menstruation, (iii) chronological age ≥ 20 years at conception, and (iv) single-fetus gestation. In addition, the following exclusion criteria were applied: (i) chronic diseases before pregnancy (systemic arterial hypertension, diabetes mellitus (DM), and human immunodeficiency virus infection); (ii) active syphilis requiring treatment, celiac disease, lactose intolerance, and muscle lipodystrophy; (iii) use of psychoactive medications; and (iv) adherence of restrictive diets (e.g., vegetarian and lacto-ovo vegetarian).

To form the sample of the present study, we selected pregnant women from the database who answered a semiquantitative food frequency questionnaire (FFQ) at the fourth consultation with the nutritionist, during the third trimester of pregnancy. To ensure data reliability, a trained, standardized, and supervised team performed all data collection procedures.

The data collection procedures included interviews with the nutritionist and analysis of the medical records of pregnant women (until puerperium) and their newborns. Each information was recorded in its own form.

Food intake was evaluated using the FFQ administered by trained interviewers at the fourth prenatal consultation during the third trimester of pregnancy, as previously reported.

**Figure 1:**
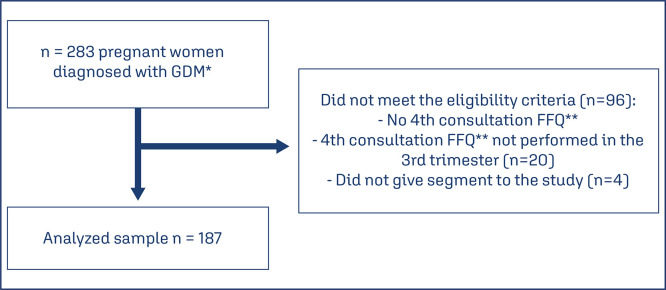
Study populaton selection criteria *GDM – gestational diabetes mellitus; **FFQ – food frequency questionnaire

The FFQ was initially composed of 20 food items: milk, cheese, butter, beef, chicken, egg, fish, beans, rice, pasta, bread, vegetables, fruits, sugar, sweets/chocolates, soda, fried foods, mayonnaise, oil, and sweetener (other foods recorded in the questionnaire may be included). The individual consumption frequency was transformed into daily frequency, assigningthe following values: 1 time/day = 1; 1 time/week =0.14; 1 time/fortnight = 0.07 and 1 time/month =0.03, never =0. The daily portion (grams/milliliters) of each food was calculated by multiplying theportion consumed and daily frequency. In this way, at the end of the detailed analyzes of all QFCA, a total of 198 food items that were grouped into 16 food groups according to similarity in composition and nutritional properties and the foods consumed by more 60% of the studied sample were not grouped with other foods and there was no overlapping. Initially, the sample consisted of 283 pregnant women. Of them, 96 women did not meet the eligibility criteria. Thus, the final sample included 187 adult pregnant women with GDM and their newborns (Figure 1).

The database was constructed mainly based on the Brazilian Table of Food Composition^13^ and the table of homemade measures.^14^ Food items not found in these tables were searched from scientific articles, food labels or packaging, and the Household Budget Survey.^15^ Standardized recipes were developed for preparations that were not found in the search, and the nutritional composition of the food items were subsequently investigated. For common industrialized foods, usually not enriched, the values of the foods labels were used as the average of several brands.

The DPs were extracted using reduced rank regression (RRR). This method has been used in nutritional epidemiology since 2004; however, few published studies to date have used this technique in relation to the topic of the present study.^16^

RRR is a hybrid approach because it considers a priori knowledge about the relationship between food consumption and the outcomes of interest and the a posteriori exploratory analysis of the data obtained through the evaluation of food consumption.^16^ In this sense, RRR explains the variability of two groups of variables, which, in this study, were the predictive variables represented by the dietary groups and the response variables represented by carbohydrate density (daily carbohydrate intake in grams divided by total daily energy intake in kilocalories), fibre density (daily dietary fibre intake in grams divided by total daily energy intake in kilocalories), and density of saturated fatty acids (daily intake in grams divided by total daily energy intake in kilocalories), due to its relationship previously described in the literature with the outcomes of interest, BW and weight adequacy for GA.^17,18^

Thus, this technique was used to identify DPs that have the potential to be associated with some health outcomes.

Height and current gestational weight were measured at the first prenatal consultation by nurses in the outpatient clinic of the unit.^19^ The pregestational weight was reported by the pregnant women or measured up to the 14th gestational week. The women were classified according to their pregestational body mass index (BMI).^20^ The adequacy of total gestational weight gain was evaluated according to the recommendation of the Institute of Medicine,^21^ validated for Brazilian women.^22^

Weight and GA at birth were collected from the medical records of the newborns and recorded in the questionnaire. BW was stratified into the following categories: low weight, < 2500 g; adequate weight, ≥ 2500 g and < 4000 g; and macrosomia, ≥ 4000 g.^23^

Weight classification for GA was performed according to the INTERGROWTH-21st project. Small for GA (SGA) was defined as BW below the 10th percentile, adequate for GA (AGA) as BW between the 10th and 90th percentiles, and large for GA (LGA) as BW above the 90th percentile.^24^

Sociodemographic information was collected, including maternal age, marital status, education level, skin colour, social habits, physical activity level classified in two categories (Do or do not do physical activity), and housing sanitation conditions (classified as inadequate in the absence of one or more of the following services: piped water, sewage network, and garbage collection).

In addition, the following clinical, obstetric, and prenatal data were also analysed: maternal age at birth; GA at birth; GA at the first visit as assessed with ultrasonography; number of pregnancies; number of deliveries; number of prenatal care visits; number of prenatal nutritional care consultations; intergestational interval; dietary guidance method (traditional method or carbohydrate counting); metabolic control (adequate glycaemic control was considerate if all the values were within the target : fasting plasma glucose level < 95 mg/dL, 1-h postprandial plasma glucose level < 140 mg/dL, and 2-h postprandial plasma glucose level < 120 mg/dL);^2^ maternal glycaemia in the third trimester; insulin use; presence of complications during pregnancy (such as: urinary tract infection, threat of premature birth, anemia, etc.), delivery, and the immediate puerperium; and delivery route.

The sample size for the current study was estimated based on the assumption that dietary analysis requires at least five subjects for each food group when the FFQ has >15 food items.^25,26^ The analysis aggregated sixteen food groups to identify the DPs during pregnancy. Therefore, the minimal required sample size was set to 80 (5 × 16) pregnant women. The number of subjects analysed in this study was consistent with the method requirements.

Initially, a descriptive analysis of the sample was performed. Categorical variables were described using absolute (n) and relative (%) frequencies, and continuous variables were expressed as mean and standard deviation.

The RRR procedure was used after standardizing the 16 food groups according to similarities in composition, nutritional properties, and percentage of consumption, to identify the DPs that possibly mediated the relationship of food intake and the perinatal results of interest in the present study. The analyses were performed using Statistical Analysis System University Edition (version 9.4).^27^

Thereafter, the DPs scores were categorized into tertiles and classified as follows: low (first tertile, T1), medium (second tertile, T2), and high (third, T3). Fisher’s exact test was used to compare the perinatal results and the DPs categories because the frequencies expected in the categories were < 5.

The associations between the tertiles of DPs and the perinatal results were estimated using multinomial logistic regression models. Relative risks (gross and adjusted) and 95% confidence intervals (CIs) were estimated to evaluate the associations between DPs tertiles and perinatal parameters (BW classification and weight adequacy for GA). The associations were considered statistically significant at p < 0.05.

Data processing and analysis were performed using Stata statistical software (version 12.0; StataCorp, 2007) and SPSS (Statistical Package for the Social Sciences) for Windows® (version 21.0). The checklist for observational studies STROBE was followed in this research.

**Figure 2:**
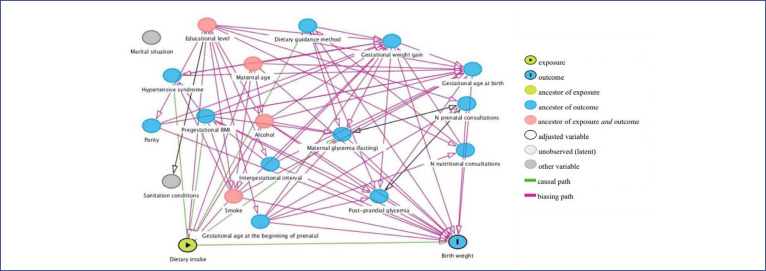
Study of dietary patterns in pregnant women with GDM and birth weight and weight adequacy for gestational age BMI – Body Mass Index; n- number

A directed acyclic graph (DAG), generated using the DAGitty program, was used to identify the confounding factors.^28^ This graph is a diagram based on causal relationships among exposure, outcome, and a set of potential confounding factors.^29^ A DAG was developed for each result, based on a priori knowledge, to identify covariates and eliminate confusion in the statistical analysis.^30^Thereafter, the covariates to be included in the statistical model of each outcome were identified as follows: current smoker (yes or no), alcohol consumption (yes or no), education level (complete or incomplete high school), and maternal age (in years) (Figure 2).

This study was approved by the Ethics and Research Committee 3.447.562 (CAAE: 15274619.2.0000.5275. All participants informed consent form at the time of data collection, after receiving explanations about all study procedures.

## Results

Most women reported having a black and other African mixed races (n = 109, 62.6%); having adequate sanitation conditions (n = 171, 93.4%); living in stable union (n = 157, 87.7%); and having completed high school (n = 99, 55.3%). With respect to maternal anthropometric characteristics, most women (n = 75, 41.2%) were overweight according to the classification of pregestational BMI, with a mean pregestational weight of 69.6 (± 19.0) kg. In addition, a predominance of total gestational weight gain above the recommendations (n = 73, 39.9%) was observed (Table 1). Data from prenatal, clinical, and obstetric care assessments showed that the mean GA, as assessed using ultrasonography at the first visit, was 11.8 (± 4.4) weeks. The mean number of prenatal care consultations was 11.9 (± 2.4), whereas the mean number of nutritional consultations was 5.7 (± 1.55). The majority of pregnant women reported being sedentary (90.7%), not drinking alcohol (91.1%) and not smoking (91.1%). During pregnancy, 70.0% of the women had good metabolic control (with glycemic targets such as described in methods) and 43.8% used insulin. The mean maternal blood glucose levels in the third trimester were 89.1 (± 12.0) mg/dL (fasting) and 116.2 (± 19.0) mg/dL (1-h postprandial). At delivery, the mean GA as assessed using ultrasonography was 38.7 (± 1.1) weeks, with the mean maternal age being 30.7 (± 5.8) years. Most of the women (67.0%) had no complications during pregnancy, delivery, and the immediate puerperium.

**Table 1:** Sociodemographic and anthropometric characteristics, data from outpatient follow-up, childbirth and immediate postpartum period of pregnant women with GDM, accompanied by the prenatal and nutrition service of a public maternity hospital

Sociodemographic and anthropometric characteristics	n	Mean	SD
Pre-gestational weight (kg)	183	69.6	19.0
GA in the first visit by the USG, in weeks	184	11.8	4.4
Number of prenatal consultations	185	11.9	2.4
Number of nutritional consultations	185	5.7	1.6
Fasting blood glucose 3rd trim. (mg/ dL )	181	89.1	12.0
Postprandial glycemia 1h 3rd trim. (mg/ dL )	166	116.2	19.0
GA at birth by USG, in weeks	175	38.7	1.1
Age at childbirth, in years	185	30.7	5.8
Number of pregnancies	185	2.4	1.4
Number of deliveries	184	1.0	1.2
Intergestational interval, in months	120	36.2	39.2
**Categorical variables**	**n(%)**		
Sanitation conditions (n=183)			
Proper	171(93.4)		
Inappropriate	12(6.6)		
Skin color (n=174)			
Black and other African mixed race	109(62.6)		
White	65(37.4)		
Education level (n=179)			
Incomplete high school	80(44.7)		
Complete high school	99(55.3)		
Marital status (n=179)			
Stable union	157(87.7)		
Single, separated, divorced, widow	22(12.3)		
Physical activity practice (n=182)			
Yes	17(9.3)		
No	165(90.7)		
Social habits (n=180)			
Alcohol	12(6.7)		
Cigarette	2(1.1)		
None	164(91.1)		
Alcohol + cigarette	2(1.1)		
BMI classification pre-gestational (kg/m)^two^			
Low weight	4(2.2)		
Adequate	52(28.6)		
Overweight	75(41.2)		
Obesity	51(28.0)		
Dietary guidance method (n=182)			
Traditional method	88(48.4)		
Carbohydrate counting method	94(51.7)		
Metabolic control (n=183)			
Lack of control	55(30.0)		
Good control	128(70.0)		
Insulin use (n=185)			
Yes	81(43.8)		
No	104(56.2)		
Adequacy of weight gain according to IOM			
Bellow	47(25.7)		
Adequate	63(34.4)		
Above	73(39.9)		
Intercurrences during pregnancy, childbirth and immediate puerperium (n=185)			
With complications	59(33.0)		
Uneventfully	124(67.0)		

SD - standard deviation; GA - Gestational Age; USG - Ultrasonography; BMI - Body Mass Index; Trim - trimester; IOM - *Institute of Medicine*

The newborns were delivered mostly via caesarean section (n = 112, 61.2%) and at term (n = 175, 95.1%). The average BW was 3261.9 (± 424.5) g, with 3.3% of the newborns (n = 6) classified as having low BW and 3.8% (n = 7) as having macrosomia. With respect to weight adequacy for GA according to INTERGROWTH-21st, 94.4% of the infants were SGA and AGA (Table 2).

**Table 2:** Characteristics of newborns for the study of dietary patterns of pregnant women with GDM and birth weight

Characteristics	n	Mean	SD
Birth weight, in grams	183	3261.9	424.5
Apgar in the 1st min	182	8.2	1.2
Apgar in the 5th min	182	8.9	0.6
Categorial variables	n(%)		
Type of delivery (n=183)			
Normal	68(37.2)		
Cesarean	112(61.2)		
Forceps	3(1.6)		
GA at delivery, in week (n=184)			
Preterm ( <37 GW)	9(4.9)		
Term (≥ 37 GW)	175(95.1)		
Newborn birth weight classification (n=183)			
Low weight	6(3.3)		
Adequate	170(92.9)		
Macrosomy	7(3.8)		
Correlation of weight for GA, according to the curve of *Intergrowth* (n=179)			
SGA	9(5,4)		
AGA	135(80,8)		
LGA	23(13,8)		

SD - Standard Deviation; Min - minute; GA - Gestational Age; GW - Gestational weeks; SGA - Small for Gestational Age; AGA - Adapting for Gestational Age; LGA - Large for Gestational Age

Three DPs were identified: DP 1 (characterized by high consumption of rice, beans, fruits and juices, and light milk and derivatives and low consumption of whole milk/foods, meat/eggs, and fish/chicken), DP 2 (characterized by high consumption of meat and eggs and low consumption of rice, fruits, and juices), and DP 3 (characterized by high consumption of refined carbohydrates, fast foods/snacks, whole milk/foods, sugars/sweets, and soft drinks and low consumption of beans, vegetables, and light milk and derivatives). DP 1 was positively correlated with fibre and carbohydrate density and negatively correlated with the density of saturated fatty acids. DP 2 was positively correlated with fibre and fatty acid density and negatively correlated with carbohydrate density. DP 3 was negatively correlated with fibre density and positively correlated with the density of carbohydrates and saturated fatty acids. In terms of the prevalence of perinatal outcomes according to the tertiles of adherence to the DPs, the highest prevalence of macrosomia was related to high adherence to the DP2 (Table 3).

**Table 3:** Prevalence of perinatal outcomes according to tertiles of adherence to dietary patterns in the 3rd trimester of pregnancy by RRR

Food patterns	n	Low adherence	Medium adherence	High Adherence	p-value
DP 1					
Birth weight					
LBW and ABW	164	53	56	55	0.63*
Macrosomy	7	3	1	3	
Adequacy of weight for gestational age^**^					
SGA	9	2	3	4	0.78*
AGA	135	46	46	43	
LGA	23	6	7	10	
DP 2					
Birth weight					
LBW and ABW	164	56	58	50	0.03*
Macrosomy	7	2	0	5	
Adequacy of weight for gestational age**					
SGA	9	2	3	4	0.21*
AGA	135	43	49	43	
LGA	23	13	5	5	
DP 3					
Birth weight					
LBW and ABW	164	54	54	56	0.16*
Macrosomy	7	4	3	0	
Adequacy of weight for gestational age^**^					
SGA	9	5	1	3	0.19*
AGA	135	41	45	49	
LGA	23	10	9	4	

*Fisher’s exact test. **Intergrowth 21- st: International Fetal Newborn and Growth Consortium for the 21st Century. DP - Dietary Patterns; LBW - Low Birth Weight; ABW - Adequate Birth Weight; SGA - Small for Gestational Age; AGA - Adequate for Gestational Age; LGA - Large for Gestational Age

In the multinomial logistic regression analysis (Table 4), no statistically significant association was found between the tertile of dietary standards in the third trimester of pregnancy and the BW or adequacy of weight for GA, even after adjustments for alcohol use, smoking, maternal age, and education level, that were the DAG’s recommendation.

**Table 4:** Associations between tertiles of adherence to dietary patterns in the 3rd trimester of pregnancy and perinatal outcomes

Dietary patterns*	Model 1 RR** (95% CI)	p-value^***^	Model 2 RR** (95% CI)	p-value***
Macrosomy****				
DP 1				
Medium adherence	-1.16 (-3.45; 1.14)	0.32	- 1.22 (-3.56; 1.11)	0.30
High adherence	0.02 (-1.63; 1.67)	0.98	0, 45 (-1.29; 2.21)	0.60
DP 2				
Medium adherence	-17.38 (-8094.13; 8059.38)	1.00	- 220.03 ( -10903.52; 10863.17)	0.99
High adherence	0.02 (-1.63; -1.67)	0.98	1.0 5 (- 0.73; 2.83)	0.24
DP 3				
Medium adherence	-0.31 (-1.85; 1.23)	0.70	-0.15 (-1.84; 1.36)	0.77
High adherence	-16.40 (-3.454.11; 3421.30)	0.99	-19.17 ( -6409.93; 6370.41)	0.99
Adequacy of weight for gestational age^*****^				
**SGA**				
DP 1				
Medium adherence	0.73 (-1.01; 2.48)	0.41	0.71 (- 1.04; 2.48)	0.45
High adherence	0.80 (-0.95; 2.54)	0.37	0.73 (-1.05; 2.52)	0.42
DP 2				
Medium adherence	0.26 (-1.57; 2.09)	0.78	0.42 (-1.43; 2.27)	0 65
High adherence	0.83 (-0.86; 2.52)	0.34	1.08 (-0.65; 2.82)	0.22
DP 3				
Medium adherence	-1.88 (-4.03; 0.28)	0.09	-1.81 (-3.99; 0.36)	0.10
High adherence	-0.90 (-2.33; 0.54)	0.22	-0.90 (-2.37; 0.55)	0.22
**LGA**				
DP 1				
Medium adherence	0.19 (-0.96; 1.35)	0.74	0.37 (-0.58; 1.52)	0.58
High adherence	0.61 (-0.47; 1.70)	0.27	0.91 (- 0.22; 2.05)	0.12
DP 2				
Medium adherence	1.10 (-2.21; 0.01)	0.05	-1.12 (-2.27; 0.02)	0.05
High adherence	-1.04 (-2.15; 0.07)	0.07	-1.04 (-2.19; 0.02)	0.07
DP 3				
Medium adherence	-0.19 (-1.18; 0.80)	0.07	-0.21 (-1.23; 0.81)	0.06
High adherence	-1.12 (-2.35; 0.10)	0.07	-1.20 (-2.46; 0.04)	0.06

DP - Dietary Patterns; ^*^The low tertile of adherence to dietary patterns was considered the reference category; ^**^RR: Relative Risk; ^***^p-value of logistic regression models; ^****^Macrosomy considered as birth weight ≥ 4000g. Low weight and Adequate weight at birth were considered reference categories. *****AGA - weight Adequate for Gestational Age, according to the *Intergrowth 21st*, was considered the reference category. SGA - Small for Gestational Age - birth weight < p10 for gestational age and LGA: Large for Gestational Age - birth weight > p 90 for gestational age, according to *Intergrowth 21st.* Model 1 has not been fitted; model 2 was adjusted for substance use (alcohol and tobacco), maternal age and educational level

## Discussion

Few studies have investigated the associations of the DPs during pregnancy of with GDM with BW and adequacy of weight for GA. Moreover, owing to the different intermediate variables, the DPs extracted using RRR, as well as the associations between DPs, BW and adequacy of weight for GA, generally differ across the existing studies.

In the present study, no significant associations were found between DPs in the third trimester of pregnancy and BW and weight adequacy for GA. Poon et al*.* also investigated the DPs of pregnant women in the same gestational period and found similar outcomes, including the lack of significant associations.^31^

The findings of the present study can be explained by the fact that the women received guidance during pregnancy from nutritionists, in addition to a multidisciplinary team composed of an obstetrician, an endocrinologist, a nurse, and other professionals according to need, in a reputable maternity service in Rio de Janeiro, and were already in the fourth nutritional consultation at the time of the analysis. Thus, although they did not follow the nutritional guidelines throughout the perinatal period and sometimes consumed non-recommended foods such as those rich in refined carbohydrates and saturated fats (e.g., fast foods, snacks, sweets, and soft drinks), they had awareness about healthy eating and its benefits for their child. Thus, despite eating non-recommended foods, their diet included healthy foods that may be associated with the adequate growth and development of their child. This type of DP may be a contributing factor to the findings of good metabolic control and absence of complications in 70% and 67%, respectively, during pregnancy, delivery, and the immediate puerperium in this study. In addition, 92.9% of the newborns presented adequate BW and only 4.6% were born LGA, which is a major concern in pregnancies associated with GDM.

Previous studies have investigated the association between DPs and outcomes related to BW and found disparate results. Coelho et al*.* analysed the association between maternal DPs in the third trimester and infant BW. In their study, greater access to snacks (stuffed biscuits, chocolate, and chocolate chips) during pregnancy was associated with higher BW of the babies (β = 56.64; p = 0.04) of adolescent women.^32^

In the study by Günther et al.,^33^ who investigated the associations between maternal diet during the onset (T0, until the 12th gestational week) and end of pregnancy (T1, after the 29th gestational week) and neonatal results, no significant association was found between energy intake or macronutrient composition of the diet and BW, SGA, and LGA. However, vegetable consumption (41.28 g per 150 g in T0, p = 0.001; 36.67 g per 150 g in T1, p = 0.001), fruit consumption (15.25 g per share in T1, p = 0.010), and diet quality, as measured using the Healthy Eating Index (39.26 g by 10 points in T0, p = 0.004; 42.76 g for 10 points in T1, p = 0.002), were positively associated with BW. In contrast, intake of sweetened beverages (10.90 g per serving in T0, p = 0.003; 8.19 g per serving in T1, p = 0.047), higher sugar intake in T0 (8.27 g per 10 g, p = 0.032), and alcohol consumption at the beginning of pregnancy (15.32 g per 1 g, p = 0.039) were inversely associated with BW. In addition, fast food intake in early pregnancy increased the chance of macrosomia (T0: odds ratio [OR], 3.14 per 250 g; 95% CI, 1.26–7.84; p = 0.014).^33^

In a cross-sectional study conducted in Chinese pregnant women, in which the RRR method was used to derive the DPs, no association was observed between a DP similar to DP 3 in the current study and BW and adequacy of weight for GA. However, significant associations were found between medium to high dietary standards (characterized by high consumption of legumes, soybean products, vegetables, meat, dairy products, eggs, and fish and relatively low consumption of wheat and oils [similar to DP 2 in the present study] in relation to high animal protein intake) and significant increases in BW (OR [95% CI], mean: 28.6 [7.1–50.1]; high: 25.2 [2.7–47.6]; p = 0.023) and BW z score (OR [95% CI], mean: 0.07 [0.02–0.12]; high: 0.06 [0.01–0.11]; p = 0.028), as well as a significant reduction in the risk of SGA (OR [95% CI], mean: 0.86 [0.77–0.97]; high: 0.81 [0.70, 0.94]; p = 0.004). In addition, a high adherence to this pattern reduced the risk of low BW by 35% (OR [95% CI], 0.65 (0.48–0.87); p = 0.005).^10^

An observational study conducted in pregnant women with GDM in Slovenia investigated the success of non-pharmacological treatment depending on pregestational weight and its association with LGA. Although the participants showed significantly improved quality of DPs during pregnancy, LGA babies were born regardless of pregestational weight, diet, and good glycaemic control.^34^

In addition, Alves-Santos et al*.* investigated the association between DPs before pregnancy and perinatal outcomes and reported that DP characterized by high intake of fast food and bullets, similar to DP 3 in this study, increased the chance of LGA in newborns (OR, 4.38; 95% CI, 1.32–14.48).^25^ Paknahad et al*.*^35^ also investigated DPs in the pregestational period and observed a positive relationship between a DP characterized by high carbohydrate content and low fat content in pregnant women with GDM, both in the crude model (OR, 12.8; 95% CI, 1.47–24.54) and in the model adjusted for maternal age, history of diseases, communicable diseases, and energy intake (OR, 15.08; 95% CI, 1.5–26.4). In terms of macrosomia (n = 19), significant associations were observed with maternal DPs, and the risk was higher with a DP rich in carbohydrates and fats (n = 9) and a DP rich in fibre (n = 9) (p < 0.01).^35,36^

Maternal food intake during pregnancy significantly contributes to fetal development; however, this association is not consistently observed, as the current study shows. Elucidating this relationship is important because of the wide-ranging negative health consequences for children in the short and long term. Kaul et al. reported that LGA is a stronger marker of the risk of overweight/obesity in early childhood than maternal DM during pregnancy. The overweight/obesity rates in childhood were higher in LGA children born to mothers with GDM or type 2 DM. Breastfeeding was associated with a lower risk of overweight/obesity in childhood in most children; however, this association was not maintained in LGA children of mothers with DM.^37^

The present study had some limitations. First, the cross-sectional design limited the interpretation to a real causal association. Second, the sample size may have restricted the power to detect significant associations with small or moderate effects. Therefore, the non-significant associations observed in this study should be considered as hypothesis generating and need to be explored in future studies with a larger sample size. Third, the use of an FFQ to estimate food intake may have underestimated or overestimated food consumption owing to recall bias or non-understanding of the issues, although the questionnaire was administered by trained interviewers to minimize these complications. This may have influenced the accuracy and qualification of the foods consumed.

With respect to the strengths of the study, the technique used to derive DPs, RRR, is robust, although only a few related studies to date have used this method. It is a hybrid approach that considered both data from the literature and data from the current research to identify DPs that have the potential to be associated with some health outcomes. Another strength lies in the use of a DAG to identify possible confounding factors. The construction of this causal diagram is a robust and infrequent technique in studies in the health field. The DAG was developed from a priori knowledge to identify a minimal but sufficient set of covariates to remove confusion from statistical analysis.

Our results suggest that the prenatal DPs, during the third trimester, of pregnant women with GDM followed by the nutrition outpatient clinic of a public maternity hospital in Rio de Janeiro, did not affect the birth outcomes defined by the BW, SGA, AGA and LGA.

## Conclusion

However, further studies are warranted to elucidate the relationship because the analysis methods and results are inconsistent across many studies. Health education aimed at emphasizing the relevance of maintaining a healthy diet during pregnancy should be implemented, especially in women with a confirmed diagnosis of GDM, to prevent or reduce unfavourable outcomes in the mother and baby. Future studies with a larger number of participants and covering the prenatal, natal, and postnatal periods are required to improve the understanding of the association between the DPs of women with GDM and the short- and long-term gestational outcomes.
